# Online Accurate Detection of Breath Acetone Using Metal Oxide Semiconductor Gas Sensor and Diffusive Gas Separation

**DOI:** 10.3389/fbioe.2022.861950

**Published:** 2022-03-08

**Authors:** Hao Dong, Libin Qian, Yaoxuan Cui, Xubin Zheng, Chen Cheng, Qingpeng Cao, Feng Xu, Jin Wang, Xing Chen, Di Wang

**Affiliations:** ^1^ Intelligent Perception Research Institute, Zhejiang Lab, Hangzhou, China; ^2^ Key Laboratory for Biomedical Engineering of Education Ministry of China, Zhejiang University, Hangzhou, China

**Keywords:** acetone, MOS sensor, breath analysis, diffusive gas separation, ketogenic diet

## Abstract

Breath acetone (BrAce) level is an indicator of lipid oxidation rate, which is crucial for evaluating the status of ketoacidosis, ketogenic diet, and fat burning during exercise. Despite its usefulness, detecting BrAce accurately is challenging because exhaled breath contains an enormous variety of compounds. Although many sensors and devices have been developed for BrAce measurement, most of them were tested with only synthetic or spiked breath samples, and few can detect low concentration BrAce in an online manner, which is critical for extending application areas and the wide acceptance of the technology. Here, we show that online detection of BrAce can be achieved using a metal oxide semiconductor acetone sensor. The high accuracy measurement of low concentration BrAce was enabled by separating major interference gases utilizing their large diffusion coefficients, and the accuracy is further improved by the correction of humidity effect. We anticipate that the approach can push BrAce measurement closer to being useful for various applications.

## Introduction

Human excretes more than 200 volatile organic compounds (VOCs) through respiration, which contains many physiological and biochemical information (Pauling et al., 1971; Phillips et al., 1999). Due to the noninvasive nature, breath analysis has been considered to be a promising method for disease diagnosis and health management (Chen et al., 2017; Dong et al., 2017; Dong et al., 2020; Chen et al., 2021). An increasing number of breath-based tests have already been extensively employed in clinical, e.g., Helicobacter pylori test (Stefano et al., 2018), etc.

Among all these VOCs, Breath acetone (BrAce), as a by-product of the fat metabolism process, has been measured to monitor ketosis in healthy and diabetic subjects (Wang and Wang, 2013; Alizadeh et al., 2019). It has been shown that BrAce level can go up to 100 ppm for individuals with ketogenic diet and fasting conditions and increase up to 1250 ppm for poorly controlled diabetes with ketoacidosis. In contrast to these high BrAce conditions, BrAce of normal healthy individuals usually ranges from only 0.5–2.0 ppm (Anderson, 2015). The BrAce fluctuation within this range has been shown to provide insight into an individual’s lifestyle, level of hunger, and efficiency of work out (Musa-Veloso et al., 2002; King et al., 2009; Španěl et al., 2011). Thus, accurate detection of low concentration BrAce will enable the management of daily diet and exercise, alerting lifestyle and preventing the onset of diseases.

The traditional methods for trace BrAce detection employ chromatography and mass spectrometry-based standard equipment (Schwarz et al., 2009; Španěl et al., 2011). However, considering cost, size, and complexity, these methods are not practical for people to monitor their fat metabolisms frequently at different places. Chemical sensors are attractive alternatives due to their small size, low cost, and easy operation (Wu et al., 2021; Liang et al., 2022). Different types of chemical sensors have been developed for BrAce detection. Colorimetric sensors have good sensitivity and selectivity (Wang et al., 2020). However, they are typically irreversible and for one-time use only, which increases the cost of frequent use. Lots of efforts have been devoted to the development of metal oxide semiconductor (MOS) acetone sensors (Shin et al., 2013; Koo et al., 2017; Cho et al., 2020; Chen et al., 2022). MOS sensors are small, sensitive, and reusable, but the selectivity issue compromises their performance in the test of complex breath samples. Although lots of works on MOS acetone sensors have been published, very few demonstrated detection of real breath samples with good accuracy, especially for online direct breath test without using a sampling bag, which is critical for the practicality and widespread adoption of the technology.

In this work, we show that online accurate detection of BrAce can be achieved using a MOS sensor *via* diffusive separation of gaseous compounds. A breath reservoir with two check valves is used for both comfort breath sampling and gas separation. Although the MOS sensor is sensitive to many compounds in the breath, interference gases like hydrogen escape from the breath reservoir faster than acetone because of larger diffusion coefficients. We optimized the diffusion time, corrected humidity interference, calibrated the device using real breath samples against a standard instrument, and demonstrated its performance under realistic application scenarios.

## Materials and Methods

### Materials and Apparatus

MOS acetone sensors based on platinum doped tungsten oxide (SB-33) were purchased from FIS, Inc. Non-rebreathing T-pieces were purchased from Vacumetrics, Inc. Teflon membranes (VE8) were purchased from Membrane Solutions, Inc. The humidity sensor (HIH-5030-001) was purchased from Honeywell, Inc.

### Device Configuration

**FIGURE 1:**
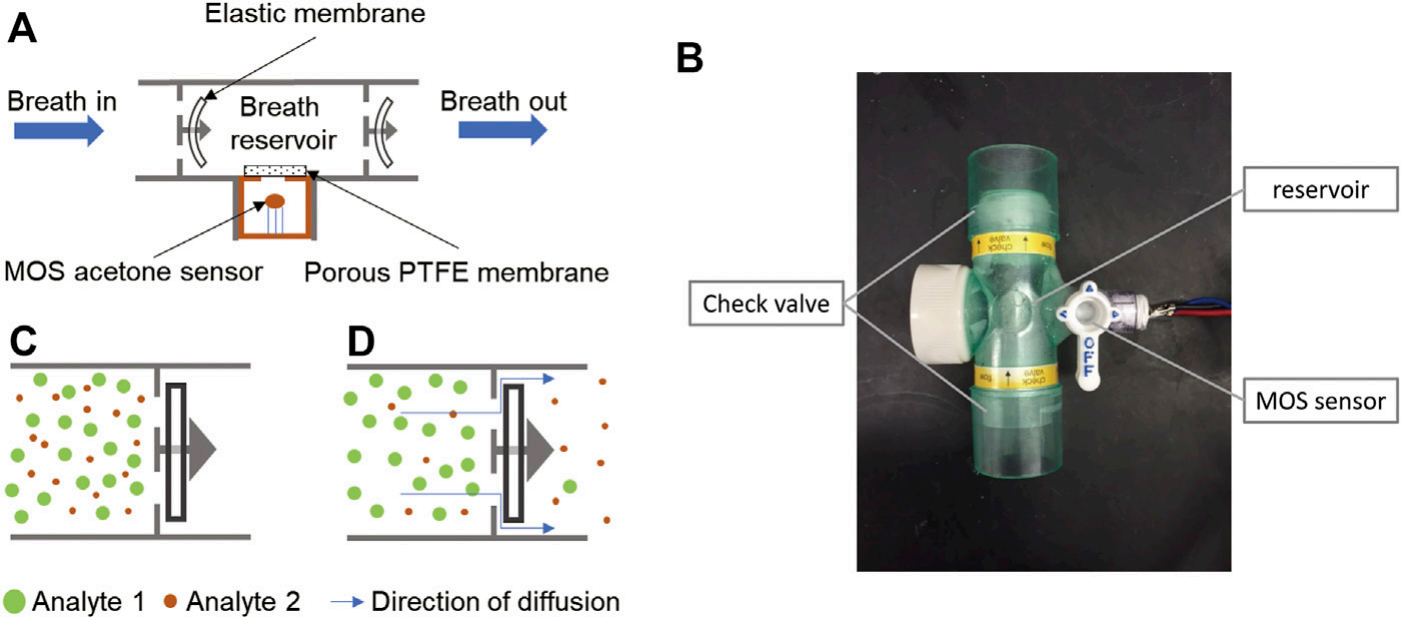
Principle and structure of the diffusive gas separation device for BrAce measurement. **(A)** During the sampling process, human subjects directly breathe into the chamber. The elastic membranes are bent by the breath pressure, allowing the exhalation to pass through. **(B)** When sampling stops, the two elastic membranes restore to their original flat shape and the breath sample is trapped. **(C)** The molecules of exhalation can escape from the reservoir *via* small gaps between the elastic membrane and its holder, while the escape speed of different molecules varied with their diffusion coefficients. **(D)** The image of the diffusive gas separation device.

The assembly diagram of the diffusive gas separation device is shown in Figure 1A, a non-rebreathing T-piece with two check valves is used for breath sampling and gas separation. The MOS sensor is placed in the reservoir for acetone sensing. Direct airflow on the sensor surface will change the conductivity of the sensor by altering the heated layer temperature. Therefore, we placed a porous Teflon membrane between the reservoir and the sensor. The membrane allows diffusion of the gases, while reducing the influence of the air speed. In addition, a humidity sensor is integrated into the reservoir for humidity monitoring and correction.

### BrAce Measurements

When the commercial MOS sensor (SB-33) is exposed to acetone, acetone reacts with oxygen ions adsorbed on the surface of the platinum doped tungsten oxide, resulting in electron transfer and the impedance change of the sensor.

The MOS sensor resistance was detected using a STM32 MCU based module integrated with an AD7792 A/D converter. We monitored the sensor baseline (R_air_) for 20 s before tests, and the response of the MOS sensor was defined as the ratio of resistance (*R*_air_/*R*).

In addition, the concentrations of BrAce were validated using a selected ion flow tube-mass spectrometer (SIFT-MS) (Instrument Science, Profile Series, Crewe, United Kingdom) in multiple ion monitoring (MIM) modes. H3O+ (m/z: 19) was chosen as the precursor ion for reaction with breath samples. The diffusive gas separation device was directly connected to the SIFT-MS injector (Supplementary Figure S1), so that a breath sample can be tested by both the acetone sensor and the SIFT-MS.

## Result and Discussion

### Principle of the Device

To collect a breath sample, a human subject directly breathes into the T-piece for about 10 s to trap the end portion of the breath. During exhalation, the elastic membranes of the check valves are bent by the breath pressure, allowing the breath to pass through (Figure 1A). After exhalation stops, the two elastic membranes restore their original flat shape (Figure 1B), which forms a breath reservoir, traps the breath sample, and prevents reverse airflow caused by inhalation. Captured gas molecules can escape from the reservoir through the small gaps between the elastic membrane and its holder (Figure 1C), and different types of gas molecules escape at different speeds because of different diffusion coefficients. Thus, the ratio of acetone concentration to interference gases changes over time. By optimizing the diffusion and measurement time, acetone can be detected with improved accuracy.

### BrAce Measurement and Interference Analysis

**FIGURE 2:**
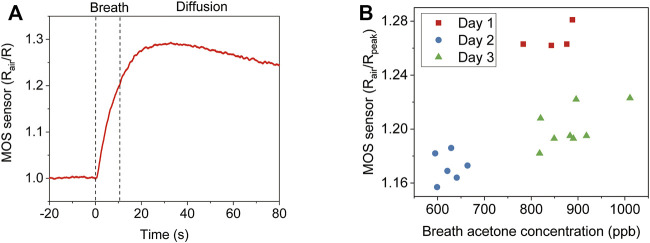
Direct analysis of breath acetone using the peak response of a MOS sensor. **(A)** The MOS sensor signal of one online breath test. **(B)** Comparison of the MOS sensor peak response and SIFT-MS analysis to the breath samples of one subject for 3 days.

Figure 2A shows the signal of one online breath test. The MOS sensor responds immediately after exhalation starts. After the exhalation stops, it takes some additional time for the gas molecules to diffuse to the MOS sensor before peak response is reached. Because the gas molecules keep diffusing into the ambient air, the signal decreases after the peak response. We further conducted three consecutive real breath tests in Supplementary Figure S2, which showed a good consistency. An intuitive way of signal analysis is simply using the peak value. To evaluate the performance, we tested the breath samples of one subject for 3 days. A selected ion flow tube mass spectrometer (SIFT-MS) was used as a reference instrument. As shown in Figure 2B, there is a clear offset between day 1 and day 3. Since no alcohol was consumed, and the ambient humidity was similar for the 3 days, other interference gases in exhaled breath can be the cause of the offset.

**FIGURE 3:**
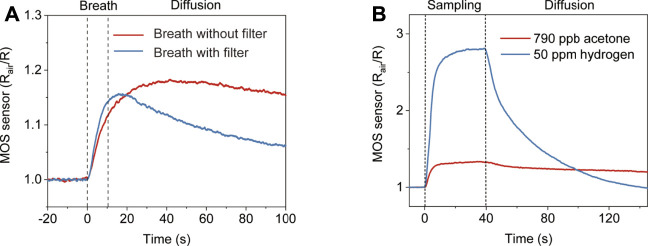
The response of the diffusive gas separation device to compounds with different diffusion coefficients. **(A)** The response of the device to two consecutive breath tests with/without the water filter. **(B)** The response of the device to 790 ppb acetone with larger diffusion coefficients and 50 ppm hydrogen with smaller diffusion coefficients, respectively.

To verify that the MOS sensor responds to other gas components in exhaled breath, a gas washing bottle filled with water was connected to the inlet to filter out acetone in breath. The removal of acetone by the bottle filter was verified by SIFT-MS (Supplementary Figure S3). Figure 3A shows the results of two consecutive breath tests, one with a filter and one without. It can be observed that the MOS sensor showed a significant response to the filtered breath. The peak response shows up earlier and the signal decreases faster than the unfiltered breath, which indicates that the interference gases in the filtered breath have large diffusion coefficients. Hydrogen is probably the major interference component, which is not absorbed by water and has a large diffusion coefficient compared to acetone. Supplementary Figure S4 shows the cross-sensitivity of the used MOS acetone sensor. Although it is less sensitive to hydrogen than acetone, the hydrogen concentration in breath can increase up to 50 ppm. Hydrogen interference is a major issue not only because of its high concentration in exhaled breath but also because its presence is hard to control by diet like breath alcohol. We solve the hydrogen interference issue by using diffusive gas separation (Figure 3B). 790 ppb acetone and 50 ppm hydrogen were prepared representing a normal BrAce level and a high breath hydrogen level. The two samples were pumped through the T-piece separately. Although the MOS sensor’s response to 50 ppm hydrogen was much higher than 790 ppb acetone, the response of the MOS sensor to hydrogen decreased much faster than acetone after sampling stopped. At 130 s, almost no hydrogen can be detected, but the response of the MOS sensor to acetone is still about 60% of the peak response. In addition, we further confirmed the diffusion coefficients of acetone-air (0.124 cm^2^s^−1^) and hydrogen-air (0.627 cm^2^s^−1^) under the circumstance (*p* = 101.325 kPa, T = 293.15 K) (Marrero and Mason, 1972).

**FIGURE 4:**
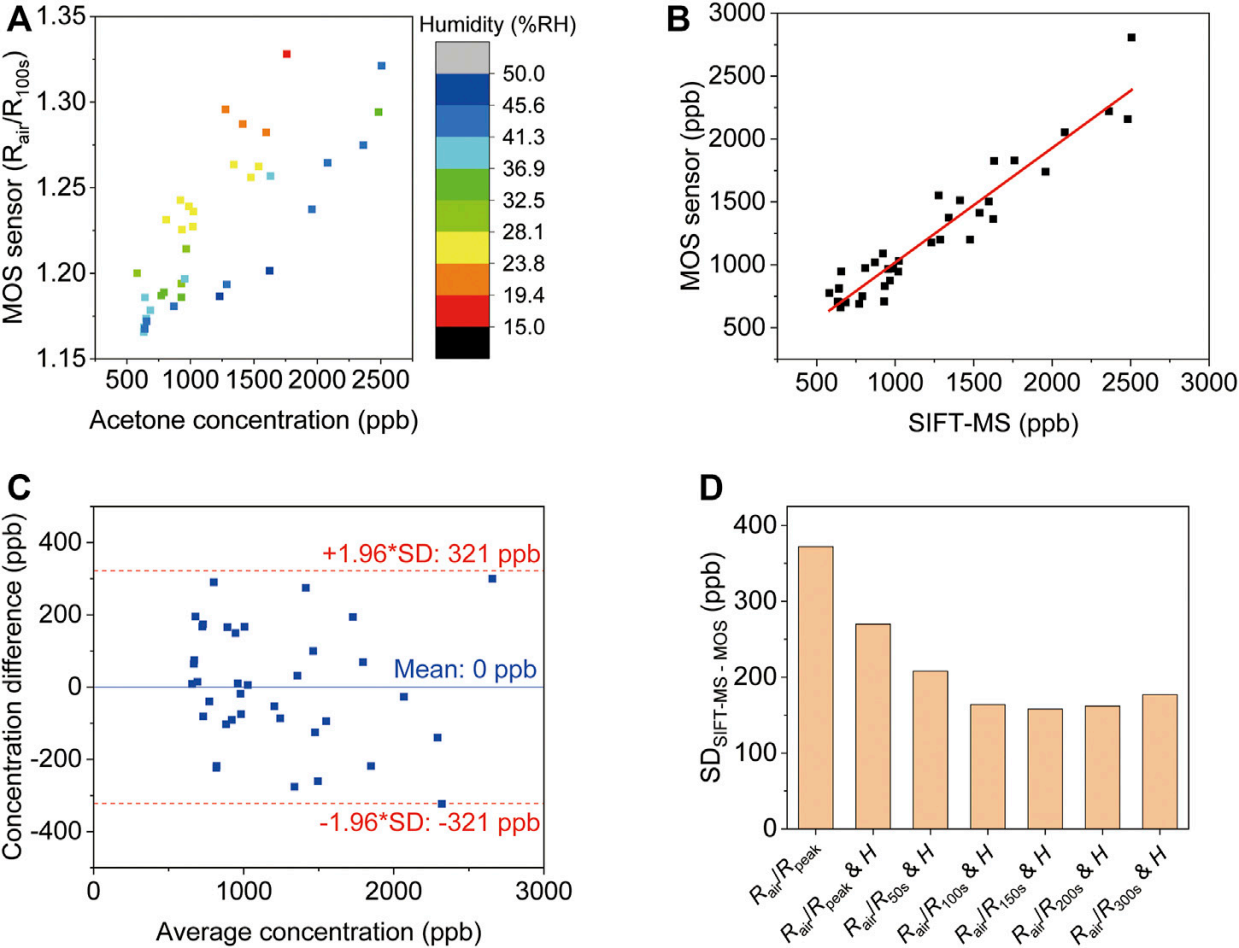
Analysis of breath acetone using the diffusive gas separation device. **(A)** The MOS sensor signals of all sample tests at 100 s (0 s indicates the start of breath sampling). The different color of the data points denotes the initial humidity level in the reservoir. The acetone concentrations of the samples were evaluated *via* SIFT-MS. **(B)** The linear correlation between MOS sensor response corrected using Eq. 1 and the breath acetone concentrations (*R* = 0.95, *p* < 0.001). **(C)** The limit of agreement between the device and the SIFT-MS. **(D)** The standard deviation of the difference between SIFT-MS and MOS using different correction parameters.

Conventionally, MOS acetone sensors are calibrated and tested using synthetic gas samples, but this doesn’t translate into accurate acetone detection in real breath samples, considering the complex components in exhaled breath. To calibrate the device, 37 real breath samples were tested in the same way as real application scenarios, i.e., directly breath into the device for 10 s. Humidity interference is another common issue for gas sensing. Although the humidity level in the reservoir is always 100 %RH after breath sampling, it can be different before each test, so a humidity sensor was placed in the reservoir to record the initial humidity level. The acetone concentration of each breath sample was confirmed by SIFT-MS. Figure 4A shows the MOS sensor signals of all the tests at 100 s (0 s indicates the start of breath sampling). The color of each data point represents the initial humidity level in the reservoir, which clearly has an impact on the response of the MOS sensor. A multiple linear regression equation was used to predict acetone concentration:

$$\left[Acetone\right]=A_{1}+A_{2}\times \left(\frac{R_{air}}{R_{100s}}\right)+A_{3}\times H$$ (1)

Where [*Acetone*] is the acetone concentration, *R*_air_ is the resistance of the MOS sensor before breath sampling, *R*_100s_ is the resistance of the MOS sensor 100 s after breath sampling starts, and *H* is the initial humidity level. Figure 4B shows that the BrAce concentrations obtained from our device and the SIFT-MS have a strong correlation (*R* = 0.95, *p* < 0.001). The best fitting values of A1, A2 and A3 for Eq. 1 are given in Supplementary Table S1.

The limit of agreement between the device and the SIFT-MS was determined from a Bland-Altman plot as shown in Figure 4C. The difference between the readings from the MOS sensor and the corresponding reading from SIFT-MS was plotted against the average reading of SIFT-MS and the MOS sensor. The limit of agreement was ±1.96 standard deviations (SD) from the mean, which was 321 ppb. This means that within 95% confidence the reading from the MOS sensor is within 321 ppb difference from the reading obtained from SIFT-MS. Regression equations using MOS signals at different time points were compared to show the benefits of the used approach (Figure 4D). Simply using the peak response value leads to the largest variation between the MOS sensor and SIFT-MS. Including humidity signal can reduce the variation. The smallest variation is obtained using *R*_150s_ (SD = 158 ppb). This allows accurate detection of low concentration BrAce.

### BrAce Based Diet Tracking

**FIGURE 5:**
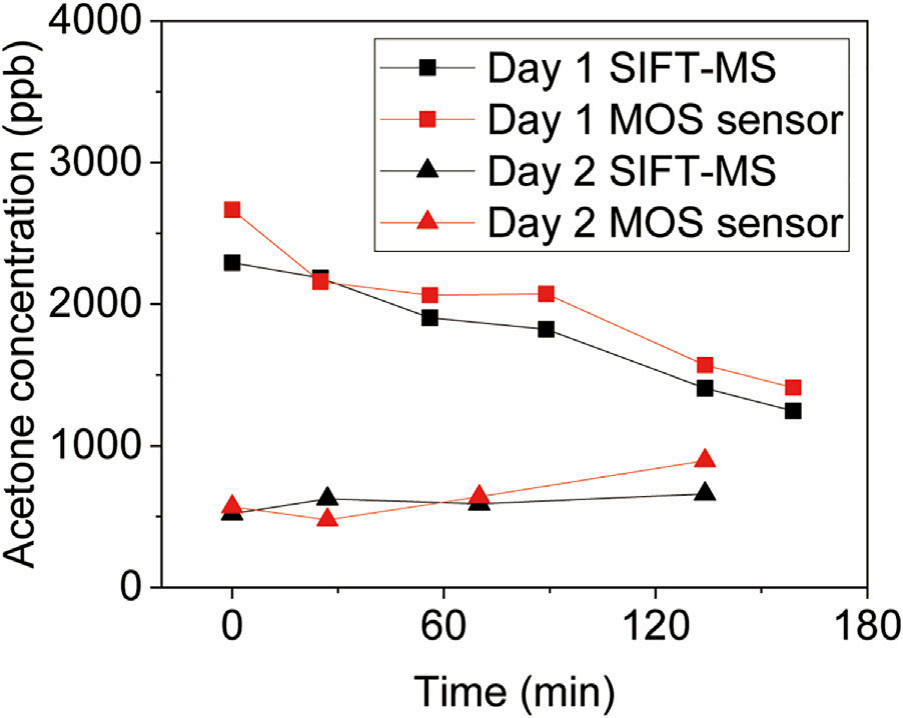
BrAce based diet tracking using diffusive gas separation device. The volunteer received a high fat and low carbohydrate dinner the day before Day 1, and a high carbohydrate breakfast about 4 h before the test. In addition, the volunteer received a normal diet on Day 2 as a comparison.

As a demo application, here we show that the device can be used to track diet (Figure 5). On day 1, the initial high BrAce level of a healthy male subject was reached *via* a high fat and low carbohydrate dinner the day before. A high carbohydrate breakfast about 4 h before the test was consumed to switch body energy source from fat to carbohydrate. It is noteworthy that eating high carb content will make bacterias in the gut produce methane from breaking down the carbs. However, the sensor (SB-33) employed in this study is much more sensitive to acetone than methane as shown in Supplementary Figure S4 in Supplementary material. As expected, a continuous decrease of BrAce was detected by both the device and SIFT-MS. A control experiment performed on day 2 shows the detection of basal BrAce under normal diet conditions. The good correlation and agreement between the device and SIFT-MS validate its reliability in the detection of real breath samples. Table 1 compares this work with recent studies of MOS acetone sensors, showing its unique feature of online breath sampling, yet with the capability of quantifying low-level BrAce.

**TABLE 1:** Comparison with recent studies of MOS acetone sensors.

Sensor	Sample	Sample delivery	Ref
Pt-WO_3_	real breath	direct exhalation	This work
PdO-Co_3_O_4_	spiked breath	air bag/pump	Koo et al. (2017)
Si-WO_3_	real breath	pump	Güntner et al. (2017)
Co_3_O_4_	synthetic gas	liquid evaporation	Zhang et al. (2015)
Ir-GO-Co_3_O_4_	synthetic gas	gas injection	Choi et al. (2014)
GO-ZnO	synthetic gas	liquid evaporation	Wang et al. (2016)
Pt-WO_3_	synthetic gas	mass flow controller	Shin et al. (2013)
Pt-PS-SnO_2_	spiked breath	air bag/pump	Jang et al. (2016)
GO-SnO_2_	real breath	air bag/syringe	Kalidoss et al. (2019)
ZnO-Co_3_O_4_	synthetic gas	syringe injection	Zhang et al. (2019)
Pt-SnO_2_	synthetic gas	mass flow controller	Cho et al. (2020)

## Conclusion

In conclusion, we proposed a simple device and novel strategy for online and accurate detection of BrAce. With a breath reservoir and two check valves, the exhaled gas can be sampled and separated in 100 s, and the influences of other interference gases in breath are significantly reduced. Moreover, we further optimized the diffusion time, corrected humidity interference, so that the device showed an excellent consistency with the SIFT-MS in real breath samples measurement. Finally, we employed the device to track BrAce of human subjects, and the results were consistent with standard instruments and can indicate different diet conditions. Compared to standard instrument-based methods, our devices showed significant advantages in complexity and cost, which enabled a simple and accurate online measurement of BrAce with conventional MOS sensors. Considering the significance of BrAce monitoring for human health, we anticipate our proposed device would promote the realization of personal monitoring of fat metabolism, provide insights into health status, and prevent various chronic diseases.

## Data Availability

The original contributions presented in the study are included in the article/Supplementary Material, further inquiries can be directed to the corresponding authors.
