# Supplementary material for: Online Accurate Detection of Breath Acetone Using Metal Oxide Semiconductor Gas Sensor and Diffusive Gas Separation
Source: Front Bioeng Biotechnol. 2022 Mar 8;10:861950. doi: 10.3389/fbioe.2022.861950 (PMC8958005; doi:10.3389/fbioe.2022.861950)
Supplement: Supplementary file 1 [file DataSheet1.pdf]

Supplementary Material for

Online Accurate Detection of Breath Acetone Using Metal Oxide Semiconductor Gas Sensor and Diffusive Gas Separation

Hao Dong[1,2], Libin Qian[1], Yaoxuan Cui[1], Xubin Zheng[1], Chen Cheng[1], Qingpeng Cao[1], Feng Xu[1], Jing Wang[1], Xing Chen[2*], Di Wang[1*]

[1] Intelligent Perception Research Institute, Zhejiang Lab, Hangzhou, 311100, China

[2] Key Laboratory for Biomedical Engineering of Education Ministry of China, Zhejiang University, Hangzhou 310027, China

*** Correspondence:** Di Wang, diwang@zhejianglab.com; Xing Chen, cnxingchen@zju.edu.cn;

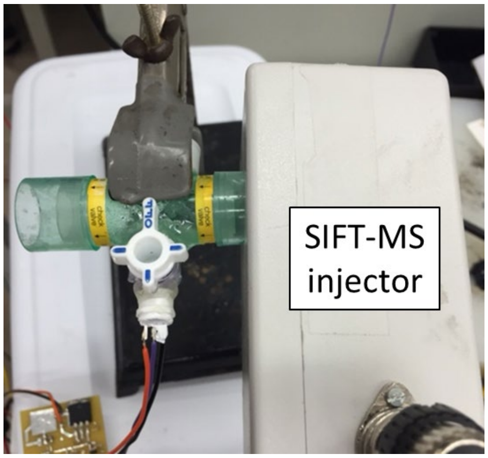

Figure S1. Illustration of using acetone sensor and SIFT to test the same breath.

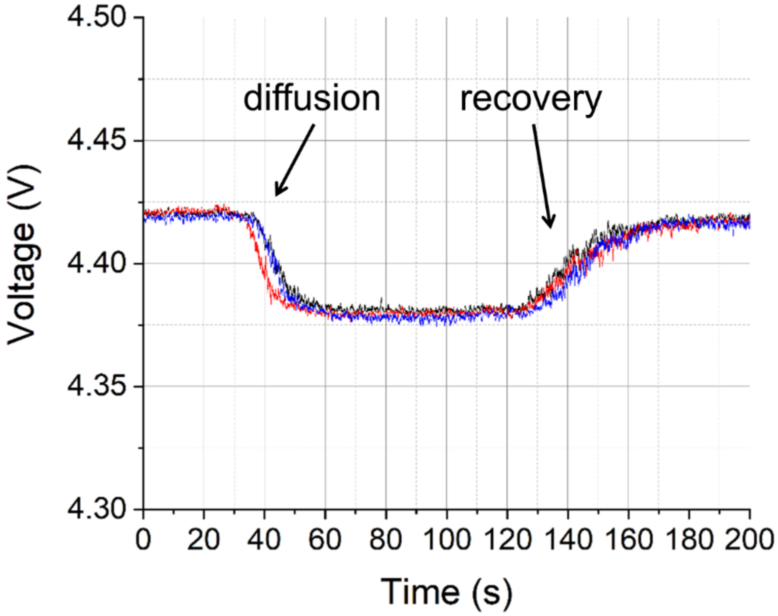

Figure S2. 3 consecutive real breath tests.

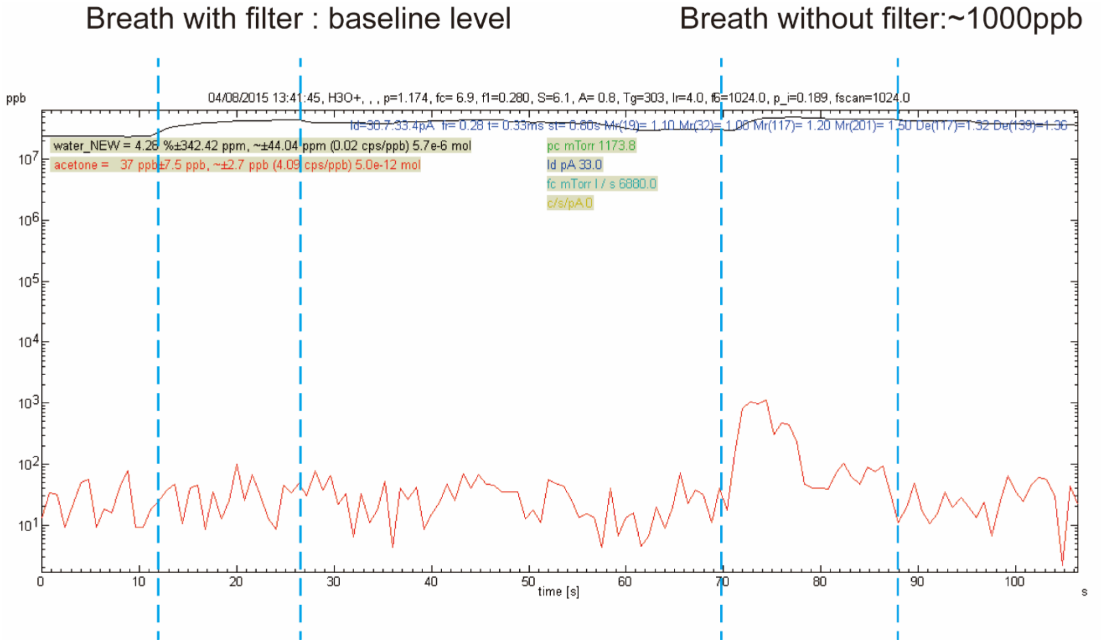

Figure S3. The SIFT-MS analysis of exhaled gas through/ not through gas washing bottle.

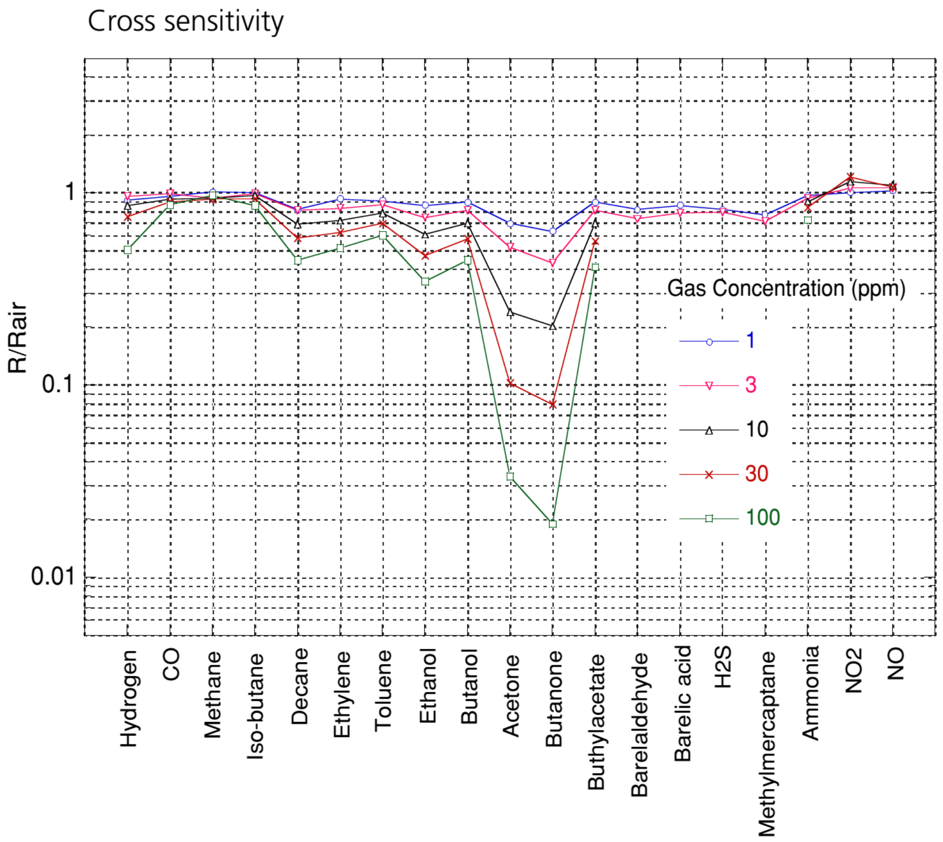

Figure S4. Cross sensitivity of SB-33 MOS sensor.

Table S1. Estimated variable values of the multiple linear regression of Equation (1).

Parameters	Estimated value	Standard error	95% confidence interval
A1	-15751	914	-17609 to -13894
A2	12732	689.9	11330 to 14134
A3	39.24	3.732	31.66 to 46.82
